# Patterns of implicit and explicit identity as a vegan or vegetarian in predicting healthy orthorexia and orthorexia nervosa

**DOI:** 10.1007/s40519-025-01734-3

**Published:** 2025-03-18

**Authors:** Ian P. Albery, Rebecca Smith, Daniel Frings, Marcantonio Spada

**Affiliations:** School of Applied Sciences, London South Bank University, London, UK

**Keywords:** Implicit identity, Explicit identity, Healthy orthorexia, Orthorexia nervosa, Perfectionism, Obsessive compulsive

## Abstract

Orthorexia nervosa (OrNe) is an eating disorder characterised by a pathological interest and preoccupation with healthy foods and a healthy diet. Evidence suggests that tendencies towards OrNe may be prevalent across diet groups, and this is particularly the case in vegans and vegetarians. Our previous work has identified that alongside individual differences in obsessive compulsiveness and perfectionism, cognitive biases (attentional preference for healthy-related cues) are associated with OrNe, whereas explicit identity (as a vegan/vegetarian) is only associated with a healthy orthorexia form. No work has assessed whether one’s known identity (explicit identity) or that form of identity which is based on fast acting cognitive associations (implicit identity) further differentiate healthy orthorexia from OrNe tendencies in addition to compulsiveness and perfectionism. One hundred and forty-four self-identified vegans (*n* = 45), vegetarians (*n* = 50) and meat-eaters (omnivores) (*n* = 49) (66 females, 74 males, 4 non-binary; *M* age = 35.09) completed measures of current hunger status, obsessive compulsivity, perfectionism, the Teruel Orthorexia Scale, perceived identity centrality as a vegan/vegetarian (explicit identity) and a “self as vegan/vegetarian” implicit association test (implicit identity). Results showed increased orthorexia tendencies in both vegans and vegetarians compared to meat eaters (omnivores) but only in terms of healthy orthorexia. In addition, no differences were shown for OrNe suggesting the diet type is not influential in pathological orthorexia. Explicit identity and current hunger status were both shown to be associated with healthy orthorexia and not OrNe. Implicit identity as a vegan/vegetarian was unrelated to both dimensions, while compulsiveness and perfectionism predicted OrNe. Despite individuals implicitly associating the self with being a vegan/vegetarian, this identity does not serve as a maker of orthorexia nervosa.

## Introduction

There is an emerging body of evidence suggesting that orthorexia, or the pursuit of a healthy diet, is best thought of in terms of both a healthy dietary interest [healthy orthorexia (HeOr)] or one that has developed into a more compulsive preoccupation [i.e., orthorexia nervosa (OrNe)] [7, 10].

HeOr is characterised as a healthy interest with a healthy diet and is independent of any psychopathology [14]. People with increased HeOr commit significant time, effort, and resources in achieving a healthy diet (i.e., the planning and preparation of healthy meals). This interest appears to be reflected as a core aspect of one’s identity [5, 25].

OrNe is thought of as “……a pathological preoccupation with a healthy diet” [10, p. 289]. Individuals are likely to be “highly concerned with and overwhelmed by their preoccupations, which lead them to negative consequences such as self-punishment, social isolation, and guilt” [10, p. 289]. OrNe has been shown to be related to a compulsive adherence to a diet based on healthy foods, strict dietary constraints, perfectionism, obsessive thoughts about healthy eating, feelings of guilt and shame after one has transgressed from only acceptable food stuffs and the avoidance of types of foods [9, 21], see [27, 28, 66, 70]). In addition, numerous negative effects of OrNe have been observed including decreased quality of life, affected interpersonal relationships and mental health indicators including obsessive compulsiveness and perfectionism [12], see [27, 50, 71]. Evidence with respect to both the association between OrNe and age [49, 60] and a gender disparity is mixed (e.g., [72]).

There is also evidence that some diet-based groups, and in particular those who have adopted a vegan (i.e., exclusively plant-based) or vegetarian (plant-based but also including eggs and diary) diet, are more likely to be over represented among those with increasing OrNe tendencies (e.g., [13, 17, 18, 26, 45]). One argument for this observation is that embracing of a vegetarian or vegan diet affords a socially conventional way to conceal an underlying pattern of disordered eating [11, 26]. A second argument suggests that the dietary restriction needed in a vegan/vegetarian diet may be just the result of the removal of meat from one’s diet and not dietary restrictions per se [78]. While our earlier work (i.e., [5]) highlighted the relationship between healthy orthorexia (HeOr) and orthorexia nervosa (OrNe) with the centrality of one’s identity as a vegan/vegetarian and an attentional preference for healthy food-related stimuli (see also [3]) among individuals who adhered to a vegan or vegetarian diet, we did not determine any differences between vegans and vegetarians in terms of experienced identity and other markers associated with OrNe tendencies (i.e., compulsiveness and perfectionism). Nor did our previous work explicitly contrast between vegans and vegetarians and people who are omnivores (those who eat meat as well as vegetarian diets). The current study examined such differences in effects between vegans and vegetarians and, in addition, included a control group of current self-identified meat eaters (omnivores). In line with previous argument (e.g., [27]), we predicted that vegans and vegetarians would show a greater tendency towards both healthy and unhealthy orthorexia versions as well other relevant indicators (i.e., compulsiveness and perfectionism) compared to omnivores.

More recently a limited number of studies have begun to explore social and cognitive processes that may be influential in the experience of elevated OrNe tendencies (e.g., [3, 5, 60, 74]). For example, evidence suggests that increasing tendencies towards OrNe behaviour is associated with an elevated concern for food-related stimuli in terms of an increased attentional bias towards such cues [3, 5]. This effect was highlighted using two different measures of OrNe-related tendencies (i.e., ORTO-15 and the Teruel Orthorexia Scale [TOS]) and two attentional bias methods (i.e., the modified Stroop [3] and the dot probe tasks [5]) in a general sample and among vegans and vegetarians. These findings are consistent with that reported for other health behaviours including drinking (e.g., [1]), cannabis use (e.g., [69]), gambling [8] and those related to eating (e.g., [2, 43]). Importantly, in utilising the TOS to differentiate healthy from unhealthy orthorexia, Albery et al. [5] showed that the operation of this bias is only relevant in describing problematic orthorexia tendencies (OrNe) and not a more healthy version (healthy orthorexia, HeOr) associated with an interest in and adherence to healthy eating. In addition, it was shown that this attentional bias was characterised by participants’ inability to effectively disengage their attention from healthy-food cues once encountered [5]. Albery et al. [5] argued that one’s inability to disengage from attending to healthy-related food stimuli may be the result of an initial threat-based processing of unhealthy cues. In other words, not disengaging from healthy-food cues may act as a buffer to any arousal experienced from the initial processing of unhealthy food cues.

A second factor suggests that one’s social identification with being an ingroup member may be important *not* in predicting OrNe but is for accounting for the healthy version (see [5]). In a group of vegans and vegetarians, increasing HeOr tendencies were predicted by an increasing identity as a vegan/vegetarian [5]. More specifically, increasing saliency (centrality) of one's social identity for oneself as a vegan/vegetarian was shown to be independently explanatory of rising HeOr—but not the more pathological orthorexia nervosa category.

This identity-based perspective argues that the motivation to align ourselves with chosen ingroups (i.e., ‘us vegans’) enables the reinforcement of our understanding of self-concept. This invested or desired affiliation with the groups we value, connect with, and share understandings with relative to other outgroups influences our belief sets and ongoing behaviours (see [44, 76]). These identity processes have also been shown to be increasingly important in explaining patterns of numerous health-related behaviours and their frequency (e.g., social media use [4]; alcohol consumption [34, 35, 47, 56]; smoking [64]) including eating behaviours and disorders (e.g., [20], see [24] for review [31, 79]). Identity processes have also been implicated in one’s likelihood to successfully change these types of behaviours (e.g., [19, 32, 33, 38, 62, 63]). In terms of vegan/vegetarianism, self-identification as a vegan or vegetarian has been proposed to act beyond a label related to one’s own food preferences to a core (or central) affiliation with one’s group that affects our expressed values and beliefs associated with the self [68]. In other words, identifying as a vegan/vegetarian is central to one’s self-concept and the strength and saliency of this identity will influence behavioural decisions because of a significant desire to identify as a member (see [32, 33, 38]). Indeed, veganism can be perceived as a set of life choices around food, clothing, etc., which imply a complex set of norms, collective experience, and personal and political stances (see review on veganism as identity by [80]).

Importantly, recent work exploring the effects of identity on these behaviours has argued that the experience of “self as a [insert ingroup member, e.g., vegan, drinker, smoker, gambler]” may be dependent on how relevant information associated with this evaluation is derived. Put simply, it has been consistently shown that social behaviours can be generated from cognitive and affective activity that we are either aware of (i.e., explicit processing: reflective, slow, deliberative) and/or *not* aware of (i.e., implicit processing: reflexive, automatic and spontaneous in nature) (see [75, 81, 82]). This dual process perspective has been applied for understanding appetitive behaviours (see [16, 29, 40, 58]). Evidence has accumulated to suggest that established problematic habitual behaviour is *less* controlled by cognition that is reasoned, deliberative and reflective (the explicit system—system 2) and more by processes (e.g., attentional processing, approach-avoidance biases, etc.,) that are automated, fast acting and more intuitive in nature (the implicit system—system 1) [58, 81, 82].

Recent work is also accumulating to suggest that the operation of (social) identities in addictive behaviours is *not* an exception to these observations. For example, theoretically, the Social Identity Model of Cessation Maintenance specifically identifies that both intuitive and deliberative cognition characterise the expression of one’s social identity in ongoing behavioural patterns associated with changing health-related behaviours [32, 33]. In addition, that aspects of one’s more conscious identity is associated with increased behavioural patterns is well-defined (e.g., [4, 5, 19, 37, 38]). For example, in examining the influence of identity factors associated with the identity dimensions of self-definition or self-investment, Albery et al. [4] showed that variability in problematic Facebook use was predicted by how chronically salient one’s group membership (a self-investment component of identity expression) was for the self (i.e., identity centrality) (see also [61]). A similar pattern was also shown for predicting increasing levels of self-reported healthy orthorexia and not orthorexia nervosa [5] as well as the severity of other negative health behaviours and their cessation maintenance in smokers [64], drinkers [19, 34–36, 46], gamblers [6, 48, 67, 73], cannabis users [15] and those recovering from eating disorders [63]. Turning to work that has examined the effects of implicit identity expression, a similar pattern has emerged (see [56]). Most of this work has concentrated on alcohol use and has typically shown heightened implicit associations of the self and being, for example, a drinker with various indicators of alcohol use and misuse cross-sectionally and prospectively (e.g., [15, 23, 34, 42, 53–55, 57, 59].

From this brief overview, while the importance of identity (both implicit and explicit) has been established for several health behaviours (e.g., alcohol consumption, smoking, gambling, social media, etc.), very little work has directly addressed the effects of identity per se on eating behaviour and in particular disordered eating (see [5] and [63] for exceptions). Of note is the lack of any studies directly assessing the strength of both implicit and explicit social identities on either general disordered eating indicators and, in particular, HeOr and OrNe behavioural tendencies. In light of this discussion, the current study examines the influence of explicitly and implicitly experienced social identities on healthy and unhealthy dimensions of orthorexia tendencies in vegans and vegetarians alongside both context specific (i.e., experienced in-the-moment hunger) and more stable factors known to be associated with either HeOr (i.e., OrNe scores) and OrNe (i.e., HeOr scores, compulsivity, and perfectionism). We predict that the *explicit* reporting of one’s identity as a vegan/vegetarian is more likely to be associated with the nonpathological healthy orthorexia (HeOr) version and not the pathological version (i.e., orthorexia nervosa [OrNe]), replicating Albery et al. [5]. Contrastingly, in addition to individual differences in compulsivity and perfectionism the experience of one’s identity as being implicitly generated is more likely to be associated with OrNe characterised as being more compulsive and habitual in nature. Put simply, we hypothesise a dissociation between healthy orthorexia and orthorexia nervosa based on the implicit or explicit expression of one’s identity as a vegan/vegetarian.

## Methods

### Participants

A total of one hundred and forty-four (66 [45.8%] females, 74 [51.4%] males, 4 [2.8%] non-binary; *M* age = 35.09, *SD* = 12.82, range = 18–63) self-identified vegans (*N* = 45; 22 [48.9%] females, 22 [48.9%] males, 1 [2.2%] non-binary; *M* age = 36.53, *SD* = 13.85, range = 18–62), vegetarians (*N* = 50; 25 [50%] females, 23 [46%] males, 2 [4.0%] non-binary; *M* age = 33.78, *SD* = 11.58, range = 18–63) and meat eaters (*N* = 49, 19 [38.8%] females, 29 [59.2%] males, 1 [2.0%] non-binary; *M* age = 35.10, *SD* = 13.15, range = 18–61) took part in the study.^1^ Participants were recruited from a research participation scheme run by the host university in exchange for course credit (*N* = 60) or opportunistically by advertising and posting the study link via researchers’ social media networks sites (*N* = 84).

Assuming 80% power, a medium Cohen’s *f*^2^ effect size of 0.14 for predicting orthorexia with four predictors (including identity as a vegan/vegetarian) (based on [5]), and an alpha is 0.05 a sample size total of 91 vegans/vegetarians was required and 95 successfully recruited.

### Design

A cross-sectional correlational design was used to test the association between both healthy and unhealthy orthorexia-related tendencies (i.e., HeOr and OrNe) and degree of identity as a vegan/vegetarian expressed implicitly or explicitly, obsessive compulsiveness, perfectionism, and in situ hunger experienced.

### Materials

#### Demographics

Age (years) and gender (male, female, non-binary) were initially recorded. In addition, all participants were asked “[D]o you identify as a vegan/vegetarian/meat eater? (yes, no for each response).^2^

#### Orthorexia nervosa

Participants completed the Teruel Orthorexia Scale (TOS; [10]) comprising 17 items measuring the dimensions of healthy orthorexia (HeOr) (nine items, e.g., “I feel good when I eat healthy food”) and orthorexia nervosa (OrNe) (eight items, e.g., “I feel guilty when I eat food that I do not consider healthy”). Responses were made on a four-point Likert scale (0 = ‘completely disagree’ to 3 = ‘completely agree’). In the current study Cronbach’s alpha for the HeOr subscale was α = 0.85 and for the OrNe subscale α = 0.84.

#### Explicit identity centrality as a vegan/vegetarian

The centrality subscale of the multicomponent in-group identification scale (see [51]) was used. Participants responded to three statements on a seven-point Likert type scales (1 = ‘strongly disagree’ to 7 = ‘strongly agree’): “The fact that I am a vegan/vegetarian is an important part of my identity”, “I often think about the fact that I am vegan/vegetarian”, and “Being a vegan/vegetarian is an important part of how I see myself”. Higher scores relate of increased of identity centrality. In the current study the Cronbach α = 0.92.

#### Implicit identity as a vegan/vegetarian

The drinking identity Implicit Association Test (IAT) [23, 34, 35, 52] was adapted for use in the vegan /vegetarian population to create the Vegan/Vegetarian Identity IAT (IAT–VVID). The IAT–VVID measured respondents’ associations for “Vegan/Vegetarian” + “Me” (& “Meat Eater” + “Not Me”) [congruent responses] versus “Vegan/Vegetarian” + “Not Me” (& “Meat Eater” + “Me”) [incongruent responses].

Initial pilot work required six participants who self-identified as vegan/vegetarian to list as many words as possible in 2 min which they thought were representative of the category vegan/vegetarian, and six participants who self-identified as meat eaters to report as many words as possible which they thought were representative of the category meat eater as they could in 2 min. For an attribute to be included in the IAT–VVID not less than two-thirds of relevant respondents (4 from 6) listed a word. From this process the identified attribute stimuli to be included in the IAT–VVID for the “Vegan/Vegetarian” category were *green*, *healthy*, *environment*, and *lifestyle* and for the “meat eater” category were *meat*, *steak*, *animals*, and *carnivore*. Attributes for the “Me” and “Not Me” categories were derived from earlier work (e.g., [52]) utilising the drinker identity IAT (i.e., “Me”: *self*, *me, mine, my*), “Not me”: *they, them, their, other*).

The IAT–VVID contained seven blocks of trials. Three blocks were designed to familiarise participants with the task requirements, the attribute stimuli, and the sorting guidelines (block 1 [20 trials], block 2 [20 trials] and block 5 [20 trials]) (see [41]). In the critical trial blocks (block 3 [20 trials] and block 4 [40 trials] and block 6 [20 trials] and block 7 [40 trials]) participants were asked to sort attribute stimuli according to the four concepts of the IAT–VVID (i.e., Vegan/Vegetarian, Meat Eater, Me, Not Me) as quickly and as accurately as possible by pressing either a left (i.e., ‘f’ on the response keyboard) or right (i.e., ‘j’ on the response keyboard) button to indicate the location of the relevant category. For instance, in one variation stimuli belonging to “vegan/vegetarian” or “me” categories were sorted using the key on the left and stimuli belonging to the “meat eater” or “not me” categories were sorted using the key on the right (i.e., congruent trials). In this instance, after completing two blocks (60 trials], the pairings were switched such that stimuli belonging to the “vegan/vegetarian” or “not me” categories were sorted using the left key and stimuli belonging to the “meat eater” or “me” categories were now sorted using the right key (60 trials) (i.e., incongruent trials). The order of the pairings was counterbalanced across participants.

Response latencies (ms) for critical trial blocks (i.e., separately for congruent and incongruent blocks) were recorded. IAT–VVID scores were calculated using the D-measure algorithm for built in error penalty procedure specified as good practice in Greenwald et al. [41].^3^ Positive D scores indicated an implicit positive association between self and vegan/vegetarian and negative scores an implicit association for the self as meat eater.

#### Perfectionism

The concern over mistakes subscale of the Frost Multi-Dimensional Perfectionism Scale (FMPS; [39]) was used. Participants responded to nine statements (e.g., “I hate being less than the best at things”) on a five-point Likert scale (1 = ‘strongly disagree’, 5 = ‘strongly agree’). Higher scores indicate an increased tendency to experience negative emotions, because negligible mistakes are construed as failures. Current study Cronbach’s α = 0.87.

#### Obsessive compulsiveness

The 18-item item (e.g., “I find it difficult to control my own thoughts”) Obsessive Compulsive Inventory-Revised (OCI-R; [30]) scale comprising 18 items was used. Participants marked on a 5-point Likert type scale (0 = ‘not at all’, 4 = ‘extremely’) how often they experienced a series of events during the past month. Higher scores indicate increased distress caused by obsessive–compulsive symptoms. Current study Cronbach's α = 0.90.

#### Current hunger

Prior to the completion of all questionnaires and tasks current levels of hunger was measured using two statements (i.e., “at the moment I feel hungry” and “at the moment I do not feel hungry”) on a 5-point Likert scale (1 = ‘strongly disagree’ to 5 = ‘strongly agree’) (see [77]).

### Procedure

After giving ethical consent, participants confirmed that they did not have a diagnosed eating disorder, recorded their age (years) and gender (male, female, non-binary) and whether they self-identified as a vegan (yes, no), vegetarian (yes, no) or meat eater (yes, no). Participants then rated their current level of hunger and completed the TOS. Following this, participants completed the implicit association as a vegan/vegetarian task (IAT–VVID) and the explicit identity as a vegan/vegetarian scale (EIV). These measures were counterbalanced with half completing the IAT–VVID before the EIV and the other half in reverse order. Participants then completed the concern over mistakes (FMPR subscale) and OCI-R. The study protocol was designed and carried out in accordance with the ethical guidance provided by the British Psychological Society. The University Research Ethics Panel of London South Bank University approved the study. All experimental tasks and questionnaires were programmed and presented using Gorilla software (www.gorilla.sc) and data generated between 20th January 2023 and 15th March 2023.

## Results

### Analytical framework

In a first phase of analysis differences between vegans, vegetarians, and meat eaters in implicit and explicit identity, HeOR and OrNe, OCD and perfectionism were tested using a series of one-way ANOVAs with Group (vegans, vegetarian and meat eater) as a between-participant factor. Tukey’s HSD was used to examine any post-hoc differences. Whether corrected mean millisecond responses (the D-score) for congruent and incongruent trials in the IAT for each group differed from 0 (i.e., no difference in ms responses to congruent and incongruent trial blocks) was examined using one-sample *t* tests. In a second phase Pearson’s *r* coefficients were calculated separately for meat eaters and vegans/vegetarians between both HeOr and OrNe and predictor variables (i.e., implicit identity, explicit identity, current hunger, OCD, perfectionism). Finally, two hierarchical regression analyses were undertaken to examine how implicit and explicit identity acted as determinants of HeOr and OrNe above and beyond other candidates (i.e., OCD, perfectionism, current hunger, and either HeOr or OrNe [depending upon the model]) *only* in vegans and vegetarians.

### Comparing vegans, vegetarians and meat eaters

#### Implicit and explicit identities

Results showed group differences for implicit identity (IAT D-score), *F* (2, 141) = 49.99, *p* < 0.001, η_p_^2^ = 0.42, with both vegans and vegetarians showing increased implicit associations for the self as vegan/vegetarian compared to meat eaters (*p* < 0.001) but not between each other (*p* = 0.983) (see Table 1 for means and standard deviations). One sample t tests against the value 0 (i.e., no difference in ms responses to congruent and incongruent trial blocks) showed both vegans and vegetarians to hold significant implicit associations for the self as vegan/vegetarian, *t* (45) = 6.74, *p* < 0.001, Cohen’s *d* = 1.00, and *t* (49) = 5.20, *p* < 0.00, Cohen’s *d* = 0.73, respectively, and meat eaters for self as meat eater, *t* (48) = 7.43, *p* < 0.001, Cohen’s *d* = 1.06,.

**Table 1:** Overall range, means and standard deviations for healthy orthorexia (HeOr), orthorexia nervosa (OrNe), implicit identity (IAT D-Score), explicit identity (ID Centrality), perfectionism and obsessive compulsiveness (OCD) for vegans, vegetarians, and meat eaters

Measure	Range	Vegans		Vegetarians		Meat eaters	
		*M*	*SD*	*M*	*SD*	*M*	*SD*
HeOr^1^	5–24	17.69	3.78	16.68	4.23	13.49	4.38
OrNe^1^	0–17	7.38	3.68	8.18	3.77	8.45	3.03
Implicit identity^2^	−1.07 to 1.47	0.36	0.36	0.35	0.47	−0.33	0.31
Explicit identity^3^	1–7	5.78	0.93	5.19	1.37	1.27	0.78
Perfectionism^4^	9–43	23.98	6.51	26.06	6.80	25.92	4.86
OCD^5^	18–79	30.40	7.96	33.36	10.02	29.47	0.32

^1^Teruel orthorexia scale total scores per subscale

^2^D score, implicit association test—positive D scores indicate an implicit association for the self as vegan/vegetarian and negative scores an implicit association for the self as meat eater

^3^Identity centrality mean score

^4^Concern over mistakes subscale total score [frost multi-dimensional perfectionism scale]

^5^Obsessive compulsive inventory-revised total score

Differences between vegans, vegetarians and meat eaters was also shown for explicit identity (identity centrality), *F* (2, 141) = 256.09, *p* < 0.001, η_p_^2^ = 0.78. Vegans and vegetarians showed increased identity centrality as vegan/vegetarian relative to meat eaters (*p* < 0.001). In addition, vegans showed increased identity centrality relative to vegetarians (*p* < 0.05) (see Table 1).

#### Healthy orthorexia (HeOr) and orthorexia nervosa (OrNe)

Results showed group differences for HeOr, *F* (2, 141) = 13.35, *p* < 0.001, η_p_^2^ = 0.16, with meat eaters reporting decreased healthy orthorexia scores relative to vegetarians and vegans (*p*s < 0.01). Vegetarians did not differ from vegans for HeOr (*p* = 0.45). For OrNe the effect for Group was not significant, *F* (2, 141) = 1.17, *p* = 0.312, η_p_^2^ = 0.02 (see Table 1).

Since there were no discernible differences between the vegan and vegetarian groups in terms of HeOR and OrNe scores they were combined to form a single group for use in subsequent analyses involving identity measures and OrNe or HeOr (*N* = 95).

#### OCD and perfectionism

No significant differences between vegans, vegetarians and meat eaters were found for either perfectionism, *F* (2, 141) = 1.65, *p* = 0.19, η_p_^2^ = 0.02, nor obsessive compulsiveness, *F* (2, 141) = 2.42, *p* = 0.09, η_p_^2^ = 0.03.

### Determinants of healthy orthorexia (HeOr) in meat eaters

Initial Pearson’s *r* correlation coefficients were calculated between HeOr and other variables.^4^ After Bonferroni correction (*p*s < 0.007) no variables were shown to correlate significantly with HeOR (see Table 2).

**Table 2:** Pearson’s r correlation coefficients for candidate variables included in regression models predicting healthy orthorexia (HeOr) and orthorexia nervosa (NeOr) in meat eaters

Variable	2	3	4	5	6	7	8
1. HeOr^1^	0.41***	−0.15	0.19	−0.02	0.15	−0.11	0.33*
2. OrNe^1^	–	−0.12	0.02	0.03	−0.09	0.19	0.31*
3. Implicit identity^2^		–	−0.12	−0.29*	0.10	−0.19	0.23
4. Perfectionism^3^			–	0.43**	0.00	0.16	0.14
5. OCD^4^				–	−0.12	0.56***	−0.04
6. Age					–	−0.06	0.06
7. Gender^a^						–	−0.01
8. Currently hungry^5^							–

*N* = 49; *p < .05, **p < .01, ***p < .001

^1^Teruel orthorexia scale total scores per subscale

^2^D score, implicit association test—positive D scores indicate an implicit association for the self as vegan/vegetarian and negative scores an implicit association for the self as meat eater

^3^Concern over mistakes subscale total score [frost multi-dimensional perfectionism scale]

^4^Obsessive compulsive inventory-revised total score

^5^Composite scores of currently hungry plus not currently hungry measures—increased scores represent increased hunger

^a^Gender 1 = Male, 2 = Female

### Determinants of healthy orthorexia (HeOr) in vegans and vegetarians

Initial Pearson’s r correlation coefficients showed HeOr to be significantly associated with OrNe, explicit identity as a vegan/vegetarian and in-the-moment hunger judgements^5^ (*p*s < 0.001) (after Bonferroni correction, *p* < 0.007) (see Table 3).

**Table 3:** Pearson’s r correlation coefficients for candidate variables included in regression models predicting healthy orthorexia (HeOr) and orthorexia nervosa (NeOr) in vegans and vegetarians

Variable	2	3	4	5	6	7	8	9
1. HeOr^1^	0.58***	0.02	0.36***	0.17	0.16	0.22*	−0.04	0.36***
2. OrNe^1^	–	−0.08	0.22*	0.52***	0.45***	0.02	0.14	0.12
3. Implicit identity^2^		–	0.01	−0.09	0.04	0.11	−0.03	0.03
4. Explicit identity^3^			–	0.28**	−0.07	0.23*	−0.27**	0.19
5. Perfectionism^4^				–	0.27**	−0.05	0.01	0.03
6. OCD^5^					–	−0.01	0.14	−0.02
7. Age						–	−0.39***	0.34**
8. Gender^a^							–	−0.19
9. Currently hungry^*6*^								–

*N* = 95; *p < .05, **p < .01, ***p < .001

^1^Teruel orthorexia scale total scores per subscale

^2^D score, implicit association test—positive D scores indicate an implicit association for the self as vegan/vegetarian and negative scores an implicit association for the self as meat eater

^3^Identity centrality mean score

^4^Concern over mistakes subscale total score [frost multi-dimensional perfectionism scale]; ^5^Obsessive compulsive inventory-revised total score

^6^Composite scores of currently hunger and not currently hungry measures—increased scores represent increased hunger

^a^Gender 1 = Male, 2 = Female

To examine the effects of implicit (D-score) and explicit identity (identity centrality) on HeOr controlling for the effects of OrNe and current hunger a hierarchical multiple regression analysis was performed. Current hunger was included in the step 1, OrNe added to the equation at step 2 and implicit and explicit identity added in step 3. In terms of assumptions, a sample size of 95 was adequate given four predictor variables (see [65]), the correlation between predictor variables was <0.80 and collinearity statistics were within acceptable limits indicative of low multicollinearity (Tolerances > 0.10; VIFs < 10). The calculation of Mahalanobis distance scores showed no significant multivariate outliers and residual and scatterplots showed that the normality, linearity, and homoscedasticity assumptions were met.

Results showed that in the first step current hunger significantly predicted HeOr, *F* (1, 93) = 14.15, *p* < 0.001, *R*^2^ = 0.13, Adj. *R*^2^ = 0.12, Cohen’s *f*^2^ = 0.15. Adding OrNe score to the equation also resulted in a significant effect, *F* (2, 92) = 34.14, *p* < 0.001, *R*^2^ = 0.43, Adj. *R*^2^ = 0.41, Cohen’s *f*^2^ = 0.75, and significantly increased variance explained, *F* (1, 92) = 47.11, *p* < 0.001, *R*^2^_change_ = 0.29. The addition of implicit and explicit identity at step three also showed as significant effect, *F* (4, 90) = 19.57, *p* < 0.001, *R*^2^ = 0.47, Adj. *R*^2^ = 0.44, Cohen’s *f*^2^ = 0.89. The inclusion of implicit and explicit identity added significantly more explanatory variance, *F*_change_ (2, 90) = 3.29, *p* < 0.05, *R*^2^_change_ = 0.04.

OrNe (*B* [SE] = 0.55 [0.09], β = 0.51, *t* = 6.43, *p* < 0.001, 95% CIs [0.382, 0.724], sr^2^ = 0.25), explicit identity (*B* [SE] = 0.66 [0.27], β = 0.20, *t* = 2.47, *p* < 0.05, 95% CIs [0.130, 1.19], sr^2^ = 0.04) and current hunger (*B* [SE] = 1.05 [0.32], β = 0.26, *t* = 3.32, *p* < 0.01, 95% CIs [0.423, 1.68], sr^2^ = 0.07) were shown to be significant predictors (see Table 4).

**Table 4:** Hierarchical regression statistics for predicting healthy orthorexia (HeOr) in vegans and vegetarians from current hunger, orthorexia nervosa (OrNe), implicit identity, and explicit identity

Model	Predictor	*B* [SE]	β	*t*	s*r*^2^	*R*	*R*^2^
Step 1						0.36	0.13**
	Current hunger	1.46 [0.39]	0.36	3.76**	0.13		
Step 2						0.65	0.43**
	Current hunger	1.19 [0.32]	0.30	3.74**	0.09		
	OrNe	0.59 [0.09]	0.55	6.86**	0.29		
Step 2						0.68	0.47*
	Current hunger	1.05 [0.32]	0.26	3.32*	0.07		
	OrNe	0.55 [0.09]	0.51	6.43**	0.25		
	Explicit identity	0.66 [0.27]	0.20	2.47*	0.04		
	Implicit identity	0.46 [0.74]	0.05	0.62	<0.00		

*p < 0.05, **p < 0.001

### Determinants of orthorexia nervosa (OrNe) in meat eaters

Pearson’s r correlation coefficients showed no variables to correlate significantly with OrNe after Bonferroni correction (*p*s < 0.007) (see Table 2).

### Determinants of orthorexia nervosa (OrNe) in vegans and vegetarians

Initial Pearson’s r correlation coefficients showed OrNe to be significantly correlated with HeOr, perfectionism and OCD (*p*s < 0.001) (after Bonferroni correction, *p* < 0.007) (see Table 3). To examine the effects of implicit (D-score) and explicit identity (identity centrality) on OrNe after controlling for the effects of HeOr and current hunger, and alongside perfectionism, and OCD a hierarchical multiple regression analysis was performed. Current hunger was included in the step 1, OrNe added to the equation at step 2, and implicit and explicit identity, OCD and perfectionism added in step 3.

All regressions assumptions were met. A sample size of 95 with six predictor’s variables was adequate (see [65]). The correlation between predictor variables was <0.80. Collinearity statistics were within acceptable limits indicative of low multicollinearity (Tolerances > 0.10; VIFs < 10) and Mahalanobis distance scores showed no significant multivariate outliers. Residual and scatterplots showed that the normality, linearity, and homoscedasticity assumptions were met.

At step 1 OrNe was not shown to be significantly predicted by current hunger, *F* (1, 93) = 1.39, *p* = 0.24, *R*^2^ = 0.02, Adj. *R*^2^ = 0.00, Cohen’s *f*^2^ = 0.02. At step 2 the combination of current hunger and HeOr significantly predicted OrNe, *F* (2.92) = 24.59, *p* < 0.001, *R*^2^ = 0.35, Adj. *R*^2^ = 0.33, Cohen’s *f*^2^ = 0.49 accounting for significantly increased variance,* F*_change_ (2, (1,92) = 47.11, *p* < 0.001. In the final step (step 3) the addition of implicit and explicit identity, perfectionism and OCD to the equation resulted in a significant regression equation, *F* (6, 88) = 21.72, *p* < 0.001, *R*^2^ = 0.60, Adj. *R*^2^ = 0.57, Cohen’s *f*^2^ = 1.33, accounting for a significant increase in variance explained, *F* (4, 88) = 13.57, *p* < 0.001, *R*^2^_Change_ = 0.25.

HeOr (*B*[SE] = 0.48 [0.07], β = 0.52., *t* = 6.60, *p* < 0.001, 95% CIs [0.334, 0.622], sr^2^ = 0.20), perfectionism (*B*[SE] = 0.20 [0.04], β = 0.36, *t* = 4.86, *p* < 0.001, 95% CIs [0.118, 0.282], sr^2^ = 0.11) and OCD (*B* [SE] = 0.11 [0.03], β = 0.27, *t* = 3.73, *p* < 0.001, 95% CIs [0.051, 0.169], sr^2^ = 0.06) were shown to be significant independent predictors (see Table 5).

**Table 5:** Regression statistics for predicting orthorexia nervosa (OrNe) in vegans and vegetarians from current hunger, healthy orthorexia (HeOr), implicit identity, and explicit identity, OCD and perfectionism

Model	Predictor	*B* [SE]	β	*t*	s*r*^2^	*R*	*R*^2^
Step 1						0.12	0.02
	Current hunger	0.45 [0.38]	0.12	1.18	0.01		
Step 2						0.59	0.35**
	Current hunger	−0.39 [0.34]	−0.10	1.15	0.01		
	HeOr	0.57 [0.08]	0.62	6.86**	0.34		
Step 3						0.77	0.60**
	Current hunger	−0.23 [0.27]	−0.06	0.85	0.00		
	HeOr	0.48 [0.07]	0.52	6.60**	0.20		
	Explicit identity	−0.12 [0.24]	−0.04	0.62	0.00		
	Implicit identity	−0.61 [0.61]	−0.07	1.01	0.01		
	Perfectionism	0.20 [0.04]	0.36	4.86**	0.11		
	OCD	0.11 [0.03]	0.27	3.73**	0.06		

**p < 0.001

## Discussion

### Orthorexia nervosa and veganism/vegetarianism

Previous research had argued that vegans and vegetarianism appear to be more likely to be overrepresented among those showing increased orthorexia tendencies (e.g., [17, 18]). The current study confirmed these observations by demonstrating significantly increased scores among vegans and vegetarians compared to self-defined meat eaters but only in the healthy version (i.e., HeOr). Importantly, vegans and vegetarians were equivalent in the magnitude of reported HeOr and no group differences were apparent for OrNe. This suggests that the likelihood of OrNe tendencies which may be considered increasingly pathological are equivalent among meat eaters and those who have chosen a meat free diet, but vegans and vegetarians significantly show an increased nonpathological interest in healthy foodstuffs and diet. That no differences between meat eaters and vegans and vegetarians were found for factors associated with OrNe reaffirms this observation. Vegans and vegetarians were no more nor no less likely to report increased perfectionism or obsessive–compulsive scores. To our knowledge this is the first study to directly compared tendencies towards healthy orthorexia and orthorexia nervosa among meat eaters and those who have chosen a vegan/vegetarian diet. From this evidence it seems reasonable to infer that any differences between diet-based groups is not reflected in the pathological desire to eat healthy food but one that reflects an underlying belief that consuming healthy foods will result in positive outcomes. In this way individual differences in orthorexia-related tendencies are only apparent in the context of healthy orthorexia and not orthorexia nervosa. What is not clear from this understanding is whether people transition from healthy orthorexia to the more pathological version and whether this is more or less likely depending on diet group. Adopting a more longitudinal perspective in the development of orthorexia tendencies will be required to answer this question.

Consistent with earlier evidence (e.g., [5, 10, 22]), the current study also showed a significant positive correlation between HeOr and OrNe. On this basis we included HeOr as a predictor of OrNe and OrNe as a predictor of HeOr as steps in the relevant hierarchical models. OrNe was shown to independently account for HeOr alongside current hunger experienced and explicit identity and HeOr was shown to account for significantly variability in OrNe alongside compulsivity and perfectionism. This suggests that while related OrNe and HeOr are predicted by different factors the pattern of responding based on these distinct factors might highlight mechanisms through which an interest in healthy foods and a healthy diet (HeOr) might transition to a more obsessional preoccupation (OrNe). Again, to answer this type of question would necessitate capturing transitions over time and noting which factors are explanatory at different points during transition.

This evidence also reflects the utility of incorporating the TOS for measuring two aspects of orthorexia (i.e., HeOr and OrNe), because previous work highlighting increased OrNe in vegans and vegetarians has (a) not consistently directly compared them with those having a diet that includes the consumption of meat and (b) did not utilise measures that differentiated healthy orthorexia from the pathological manifestations of OrNe. Indeed, of the 14 studies included in a recent review few had included an alternative diet-based control group (e.g., meat eaters) and none had adopted the idea that orthorexia tendencies can reflect either a more healthy interest in relevant foods, diet and behaviours *or* a more compulsive need or desire for such foods (see [17]). The argument is that scores indicative of increasing OrNe among vegans and vegetarians using measures such as ORTO-15 may conceptually mask that some of these individuals have more of a healthy interest in food stuffs as opposed to a more obsessional fixation. Future work should directly compare diet-based groups for orthorexia tendencies across numerous measures and different approaches for quantifying the spectrum of orthorexia (both healthy and pathological) to resolve this issue.

### Implicit and explicit identities

Previous research has also identified that information processing biases are important for understanding increasing orthorexia nervosa [5] and more recently that one’s identity may be important for distinguishing between a healthy version of orthorexia versus and a more unhealthy (pathological) version. Specifically, Albery et al. [5] showed that explicit identity as a vegan/vegetarian was a primary factor in describing increasing healthy orthorexia (HeOr) but was not implicated alongside increasing attentional bias for food-related stimuli in accounting for the unhealthy version. Albery et al. [5] argued that one potential reason why identity was *not* accounting for increasing orthorexia nervosa was because only those cognitions which operate more automatically and less under volitional control (i.e., more implicitly) would reflect a more pathological makeup. That attentional biases have been consistently shown for predicting orthorexia nervosa scores and not healthy orthorexia is consistent with this interpretation (e.g., [3, 5]; see [16]). In other words, it was argued that *implicit* identity, and not explicit identity, was a more likely candidate for predicting OrNe above and beyond known fundamental factors (i.e., OCD and perfectionism).

Prior to examining the relationship between identity measures and OrNe tendencies we first established differences between vegans and vegetarians with meat eaters in terms of their endorsed explicit and implicit identities. Vegans and vegetarian were shown to explicitly endorse the centrality of their identity of a vegan/vegetarian which meat eaters predictably did not. In terms of implicit identities, the pattern of associative responding highlighted by the implicit association test showed increased strength of association among vegans and vegetarians towards the self as a vegan/vegetarian, while meat eaters showed increased strength of association concordant with the self as a meat eater. The pattern of responding between vegans and vegetarians showed no differences for implicit association scores but a distinction was evident for the endorsement of identity centrality (explicit). Vegans in this case endorsed to a greater degree their explicit identity as a vegan/vegetarian relative to vegetarians while implicitly they viewed themselves as no different from each other. This suggests that merely asking vegans and vegetarians about their identity results in a differentiation in terms of how increasingly salient the identification is for vegans relative to vegetarians—vegans report increased saliency of the identity for them resulting in greater self-investment. Nevertheless, even though pilot work among vegans and vegetarians was undertaken to identify relevant attribute stimuli of the category vegan/vegetarian for inclusion in the IAT, future research should expand on this to identify whether there are subtleties in implicit response between vegans and vegetarians based on bespoke attributes associated with each category. In other words, work should consider the further generation of attributes related to being a vegan, a vegetarian, or an omnivore. This will enable the development of distinct IATs for these groups to further test the differentiation of these groups in terms of implicit identity pattern.

In addition, that there was no differentiation in terms of implicit association of the self as vegan/vegetarian suggests that vegans may “state” that their identity is obviously distinct from vegetarians (explicit identity), whereas in fact, it is functionally not in this domain (i.e., implicit identity). This highlights that what people *say* they think may not be what they think in terms of an associative framework. These findings in no way implicate individuals as merely reporting more socially desirable responses when consciously responding to statements related to their identity. Rather, we highlight that in this sample vegans and vegetarians hold significant implicit associations for the self as vegan/vegetarian which are not themselves differentiable but when asked explicitly about how central their identity was vegans demonstrated greater self-investment in their assumed identity.

That explicit identity and implicit identity were not significantly correlated with one another in either meat eaters or vegans/vegetarians suggests that, in this instance, these identities are likely to be operationally independent. However, to fully test these observations there is a need to ensure that the stimuli utilised in the IAT reflect fully the content of relevant stored sematic representations. In other words, in the current study stimuli for sorting according to the vegan/vegetarian category may not have fully reflected each diet type. Future work needs to ensure that veganism and vegetarianism stimuli are independently generated and then incorporated into the IAT for separate measurement and comparison. Moreover, given that the experienced level of one’s investment in the vegan or vegetarian identity might impact the chronicity of the identity attached (see [44]), and that this might manifest itself in both reflective (i.e., explicit) and reflexive (i.e., implicit associations), future work should include a measure of the length of time invested in the adopted identity.

### Predictors of healthy orthorexia and orthorexia nervosa in vegans/vegetarians and meat eaters

#### Implicit and explicit identities

Our next question concerned the pattern of identity responding to healthy orthorexia (HeOr) and orthorexia nervosa (OrNe) scores among vegans and vegetarians and meat eaters. Based on previous work [5], a priori we had predicted that among vegans/vegetarians explicit identity would be the best candidate for explaining increased HeOr, while OrNe would be better explained by its association with implicit identity in both vegans/vegetarians and meat eaters. Our results showed some partial support for this dissociative hypothesis. First of all, increased explicit identity as vegan/vegetarian was shown to predict increased healthy orthorexia and not orthorexia nervosa replicating our previous finding. Our analysis showed that explicit identity added significantly more explanatory variance above and beyond that provided by current hunger and OrNe scores in predicting HeOr. Second, implicit identity as a vegan/vegetarian was not associated with the more pathologically based OrNe in both vegan/vegetarians and meat eaters. Therefore, explicit identity dissociates healthy from unhealthy ON in vegans/vegetarians, while the magnitude of the implicit associations between *self and being a vegetarian/vegan* or *self and being a meat eater* implicit identities do not, disconfirming our prediction. Importantly, both increasing perfectionism and obsessive compulsiveness were also shown to predict OrNe in vegans and vegetarians confirming our previous findings (i.e., [5] but not in meat eaters. As such, despite individuals holding significant implicit associations between the self and being a vegan/vegetarian or meat eater, this increasing strength of association are not indicative of orthorexia nervosa and do not appear to serve as a marker of it. On this basis we cannot add implicit identity to other known more automated cognitive factors (i.e., attentional biases [3, 4] in the description of orthorexia nervosa-related tendencies in vegans and vegetarians.

What could account for this pattern of responding? While neither implicit associations nor the explicit expression of a vegan/vegetarian identity nor the implicit identity as a meat eater were found to account for OrNe, some scholars have argued that the expression of identity may be marked by the operation of cognitive processing biases such as attentional biases (see [32]). If this is the case, we would not necessarily expect implicit associations to directly account for increasing OrNe tendencies but rather be expressed *through* other possible mechanisms or processes. Why? Because the expression of implicit identities might manifest itself through the operation of cognitive biases (e.g., attentional biases, approach-avoidance biases, etc.) which, theoretically, could be either *self-related* or *problem-related*. With this in mind, we would predict, for example, that the increasingly embedded implicit associations we hold about being a vegan/vegetarian (or a meat eater) will result in increasing OrNe tendencies, because our identity influences the magnitude of any processing bias associated with self or problem-related stimuli in the environment and it is this relationship that determines the level of orthorexia nervosa. In other words, the operation of information processing biases is at least partially accounted for by our implicit (and explicit) identity-related associations, and it is this relationship that accounts for increasing pathological orthorexia—the relationship between implicit identity and increasing OrNe is *mediated* by the operation of cognitive processing biases. If we accept the replicated findings that OrNe tendencies appear to be characterised by an attentional preference (bias) towards healthy food-related stimuli (i.e., [3, 5], and that these biases might manifest as a function of increasing more automated identity [32], future work should directly the address whether the effect of implicit identity on OrNe is mediated by the size of related information processing biases. While theoretically, this position is defendable, no work has to date studied this pattern of relationship.

#### Perfectionism and obsessive compulsiveness

Clearly, among vegans and vegetarians those individual difference components previously shown in Albery et al. [5] to predict increasing OrNe tendencies in addition to cognitive processing biases and specifically the ability to disengage from healthy food-related stimuli, namely, perfectionism and obsessive compulsiveness, were replicated in the current study. These factors are consistently implicated in the operation of pathological orthorexia (e.g., [22]) and appear to interact with attentional biases [5] but do not relate to identity measures and, in particular, implicit identity. The current study showed that in vegans and vegetarians, while increasing OrNe was predicted by the combination of increasing perfectionism and obsessive compulsiveness neither was associated with implicit identity scores. That perfectionism and OCD predict OrNe, but not healthy orthorexia (HeOr) reaffirms the dissociation between these components of orthorexia, again reinforcing previous work (i.e., [5, 10, 22, 25]).

### Summary

As it stands, our recent work has now shown that among vegans and vegetarians increasing healthy orthorexia is characterised by one’s conscious beliefs about how salient the identity of being a vegan or vegetarian is (explicit identity) (i.e., replicating [5]). On the other hand, this study also shows that orthorexia nervosa is *not* accounted for by identity per se and, in particular, the identity-related implicit associations people hold but is by perfectionism and obsessive compulsiveness. Whether this pattern of results is consistent in diet groups other than vegans and vegetarians is yet to be fully established. While the current study included a group of self-declared meat eaters it is not possible to ascertain the magnitude of any explicit identity as a meat eater, because we utilised measures positioned to measure degree of vegan/vegetarian identity and not meat eater identity. This confirmed that meat eaters showed significantly decreased explicitly identity as a vegan/vegetarian as expected but future work should include an explicit measure of the identity as a meat eater to examine its significance in predicting healthy orthorexia. However, in terms of implicit meat eater identity, our results clearly show a significant implicit association between the self and being a meat eater among meat eaters but, as for vegans/vegetarians, this was not associated with increasing orthorexia nervosa tendencies. Whether explicit identity as a meat eater is predictive of healthy orthorexia is yet to be established.

## Data Availability

The data that support the findings of this study are available from the authors upon reasonable request.

## Footnotes

1 Groups did not differ in age, *F* (2, 141) = .543, *p* = .58, nor gender, Fisher’s exact *p* = .73.

2 10 participants identified as both a vegan and a vegetarian. Since the vegan identity necessarily subsumes vegetarianism and not vice versa those who self-identified as a vegan and vegetarian were recoded as vegans.

3 Trials in blocks 3, 4, 6, and 7 with latencies > 10,000 ms were eliminated and subsequently all participants for whom more than 10% of remaining trials showed reaction times faster than 300 ms were removed prior to D score calculation.

4 Meat eaters were asked about their explicit identity as a vegan / vegetarian and not as a meat eater to validate identity scores for vegans and vegetarians. As such no coefficients are reported for explicit identity and either HeOr or OrNe in meat eaters.

5 Whether or not participants were hungry or not was highly correlated, *r* = -.97. As such only how far participants agreed with the statement “At the moment I do not feel hungry” was reversed scored and summed with the item “At the moment I feel hungry” to generate a composite score. This was included in regression analyses to avoid effects of multicollinearity.
